# Assessment of aflatoxin M_1_ and B_1_ in some dairy products with referring to the analytical performances of enzyme-linked immunosorbent assay in comparison to high-performance liquid chromatography

**DOI:** 10.14202/vetworld.2022.91-101

**Published:** 2022-01-22

**Authors:** Raghda Mohamed Esam, Ragaa Shehata Hafez, Nagwa Ibrahim Mohamed Khafaga, Karima Mogahed Fahim, Lamiaa Ibrahim Ahmed

**Affiliations:** 1 Department of Food Hygiene and Control, Faculty of Veterinary Medicine, Cairo University, Giza, 12211, Egypt; 2 Department of Food Hygiene, Animal Health Research Institute, Giza, Egypt.

**Keywords:** aflatoxin B_1_, aflatoxin M_1_, enzyme-linked immunosorbent assay, high-performance liquid chromatography, mold, sensitivity

## Abstract

**Background and Aim:**

Aflatoxin M_1_ (AFM_1_) is a major fungal metabolite found in milk coming from aflatoxin B_1_ (AFB_1_) contaminated rations and is subsequently present in milk-based products demonstrating a serious public health hazard. This study aimed to investigate the levels of AFM_1_ and AFB_1_ in milk and some dairy products consumed widely by infants and children.

**Materials and Methods:**

This study investigated the incidence of AFM_1_ in 105 samples of processed cheese, Ras cheese, and raw milk (35 of each) retailed in the Egyptian markets. The degree of sensitivity and accuracy was evaluated using the enzyme-linked immunosorbent assay (ELISA) method followed by the estimation of the positive samples using the high-performance liquid chromatography (HPLC) with fluorescence detection. Mold count was determined in the examined samples by investigating AFB_1_ content using HPLC.

**Results:**

AFM_1_ was found in all investigated Ras cheese, raw milk, and 82.86% of the processed cheese samples with mean values of 51.05±6.19, 40.27±3.996, and 10.77±1.39 ng/kg, respectively. Moreover, there was statistically no significant difference between AFM_1_ levels in the core and crust parts of the tested Ras cheese. AFM_1_ contaminated Ras cheese and raw milk samples were 48.57% and 25.71%, which exceeded the European and Egyptian tolerance levels. Results showed an acceptable correlation between ELISA and HPLC methods with no significant difference (p>0.05). Alternatively, none of the examined samples proved to be contaminated with AFB_1_ despite the presence of mold with mean counts of 3.79±3.29, 4.39±4.34, and 4.84±4.29 log CFU/g in the examined processed cheese, Ras cheese, and raw milk samples, respectively.

**Conclusion:**

Therefore, it is urgent to regularly inspect the contamination of animal feeds with AFB_1_ and apply special measures and novel techniques to protect the feed and food from public health hazards.

## Introduction

Recently, consumer concerns about food safety have grown; thus, safety evaluation of milk is vital [1]. Recent studies on chemical contaminants have increased the awareness of the public health hazard of chemical residues, which may be present in dairy food. Among these chemicals, mycotoxins proved to be present in more than 50% of foods [2,3].

Mycotoxins are secondary metabolites produced by mold, with aflatoxins (AFs) representing the most toxic and carcinogenic, which can be found as pollutants in various foods, including dairy cattle feed [4,5]. *Aspergillus* species (*Aspergillus* *flavus*, *Aspergillus* *parasiticus*, and infrequently *Aspergillus nomius*) produce a majority of AFs in warm and humid conditions of tropical and subtropical climatic zones at a temperature range of 20-40°C with optimum growth temperature of 25-30°C and a minimum water activity of ≤0.85 [6].

So far, 18 AFs have been discovered, including aflatoxin B_1_ (AFB_1_), B_2_, G_1_, and G_2_, which are the major toxins; AFB_1_ is notoriously the most potent, as its incidence varies depending on the weather, season, geographical location, and storage conditions [7,8]. From its major metabolites, aflatoxin M_1_ (AFM_1_) is excreted in the milk after ingestion of feed contaminated with AFB_1_by 12-24 h reaching, a high level after limited days [9,10]. AFM_1_ is comparatively stable, as it is neither affected by processing (pasteurization, sterilization, and mildly acidic conditions) nor storage conditions**;** hence, it can be detected in cheese and other dairy products made from AFM_1_ contaminated milk [11,12].

The exposure of infants, teenagers, and prenatal women to the negative effects of AFM_1_ is a serious determinant for their health as it has mutagenic, carcinogenic, and teratogenic properties**.** International Agency for Research on Cancer (IARC) reclassified AFM_1_ from Group 2B (probably carcinogenic to people) to Group 1 (proven to be carcinogenic to people) [13,14].

Different methodologies with the consequence of varying sensitivity and accuracy have been indicated for the determination of AFM_1_, including thin-layer chromatography (TLC), high-performance liquid chromatography (HPLC), and mass spectrometry (MS) [15], which have excellent sensitivity and accuracy but necessitate extensive sample preparation, costly equipment, and well-trained personnel. Recently, sample screening is conducted using the enzyme-linked immunosorbent assay (ELISA) technique, which gives qualitative or semi-quantitative results, as it is simple, having hand holding validation, and equipment movability. It is mainly used in routine analysis and is reliable for large-scale sample analysis [2,16].

Several nations have established legal limits for this metabolite in milk and milk products that differ from one country to the next and are influenced by economic concerns. The European Commission regulation (165/2010) and the Egyptian standard permitted a level of 50 ng/kg for AFM_1_ in milk or processed dairy products [17,18]. This limit is one order of magnitude lower than the 500 ng/kg limit set by the United States and the Codex Alimentarius [19,20].

This study aimed to investigate the prevalence rate of AFM_1_ in processed cheese, Ras cheese, and raw milk samples randomly collected from Egyptian markets, referring to the analytical performance and accuracy of the ELISA method compared to HPLC.

## Materials and Methods

### Ethical approval

The study did not involve the use of human subjects.

### Study period and location

The study was conducted from September 2020 to March 2021. The samples were purchased from several dairy shops and supermarkets at Cairo, Giza, and El-Minia Governorates.

### Collection and preparation of samples

The study sample consists of 105 samples of processed cheeses, Ras cheeses, and raw milk (35 each). Samples were purchased from several dairy shops and supermarkets at Cairo, Giza, and El-Minia Governorates, then transported to a laboratory in a sterilized insulated icebox at around 4°C and analyzed on arrival.

Ras cheese samples were divided into two parts (core and crust) using a sterilized knife at a distance of one inch from the edge of the surface to assess a comparison between the core and crust, and then, each sample was thoroughly mixed.

The samples were analyzed using ELISA and HPLC methods to determine the correlation between the results for evaluating the accuracy and sensitivity degree of the ELISA technique. Total mold count was also estimated, and contaminated samples with mold were examined for AFB_1_ presence.

### Quantitative determination of AFM_1_ by the commercial ELISA

#### Preparation of samples

Twenty milliliters of raw milk were centrifuged at 3500× g for 10 min at 10°C. The fatty layer was aspirated, and 100 mL of the defatted supernatant was used directly within the ELISA kit to determine AFM_1_.

Five grams of finely grated cheese were mixed with 20 mL 70% absolute methanol in a tube with a cap. The mixture was extracted by shaking in a shaker for 30 min at 50°C, then, clarified by centrifugation at 3500 g for 10 min. A glass tube was filled with 2 mL aqueous phase, and a couple of milliliters of hexane were added and shaken for 10 s, centrifuged at 3500 g for 10 min. The top layer of the hexane was scraped off, and 100 mL aliquot was applied within the kit.

#### ELISA procedure [21,22]

To the bottom of each well of a microtiter plate, 100 mL antibody was added, gently mixed, and incubated at 25°C for 15 min; wells were emptied and washed with 250 mL washing buffer 3 consecutive times. AFM_1_ standard solutions (5, 10, 20, 40, and 80 ng/kg) or test samples (100 mL/well) were added in duplicate, gently mixed, and incubated for 30 min in the dark at a temperature range of 20-25°C. Wells were emptied and washed 3 times, and 100 mL enzyme conjugate was added, gently mixed, and incubated in the dark at 25°C for 15 min. Washing 3 consecutive times was done, then 100 mL substrate/chromogen was added to each well and mixed thoroughly before incubating for 15 min in the dark. Finally, 100 mL stop reagent was added to each well, and the absorbance was determined at 450 nm in the ELISA reader, using special software, RIDA^®^SOFT Win.net (Art. No. Z9996FF) (r-biopharm, Darmstadt, Germany). In line with the RIDASCREEN kit (Art. No. R1121 german.zip) (r-biopharm) guidelines, the lower detection limit was 5 ng/kg for milk.

### Quantitative determination of AFM_1_ by HPLC

#### Sample preparation and extraction

Milk samples were subjected to chromatographic analysis using the method described by Manetta [15]. The sample was homogenized and centrifuged for 10 min at 3000× *g*, then, 10 mL aqueous phase was diluted with 10 mL deionized water and purified using a solid-phase extraction-C18 carbograph-4 cartridge, which was conditioned with acetonitrile (5 mL) and deionized water (10 mL). Following the application of the diluted samples and washing with 10 mL water, 20 mL acetonitrile/water (20:80, v/v), and 10 mL n-hexane, AFM_1_ was distilled with 6 mL dichloromethane/acetone (95:5, v/v), the elute was evaporated under a gentle stream of nitrogen and the residue dissolved in acetonitrile (200^−1^); HPLC analyzed an aliquot (10^−1^) of the AFM_1_ extract.

Cheese samples were prepared using the method described by Sakuma *et al*. [23]. Briefly, 10 g of cheese was blended with 40 mL acetonitrile: methanol:water (6:1:3, v/v/v) for 10 min, before being homogenized at 4000 rpm for 5 min, then centrifuged at 3000 rpm for 5 min. Ten milliliters of the supernatant were blended with 30 mL phosphate-buffered saline (PBS; pH 7.4) before filtered using a glass filter (934AH, Whatman plc, Maidstone, Kent, UK). The Immunoaffinity column was conditioned with 10 mL PBS, then 20 mL filtrate was loaded onto the column. Finally, the column was washed with 10 mL PBS and 10 mL water. The column was eluted with 1 mL acetonitrile 3 times, and the distill was evaporated under nitrogen gas. Two hundred microliters of trifluoroacetic acid and 200 mL hexane were added to the dried distill, and the mixture was kept for 10 min at 40°C. The mixture was then allowed to dry and eventually dissolved in 1 mL acetonitrile: water (2:8, v/v); then, the solution was filtered through a 0.45 mm filter. The residue was subjected to HPLC.

#### HPLC procedure

HPLC analysis was applied using an Agilent 1260 series; Agilent Technologies, Waldbronn, Germany. The separation was performed using Eclipse C18 column (4.6 mm×250 mm i.d., 5 mm) (Waters, Milford, MA). Acetonitrile-water (25:75, vol/vol) was delivered to the column at 1 mL/min rate within the mobile phase [24]. The mobile phase utilized isocratic programming. A disposable filter unit (0.45 m) was used to filter the mobile phase. The HPLC system detected AFM_1_, using a fluorescence detector (RF 20A) at 365 nm (excitation wavelength) and 435 nm (emission wavelength). The injection volume was 10 mL and the column temperature was 40°C. The detection limit for AFM_1_ in dairy products was 0.002 mg/L [25,26].

#### Total mold count was applied according to ISO, 2012 [27]

Total mold count was estimated for all examined samples using Sabouraud’s Dextrose Agar (CM0041B Oxoid™, Belgium). The isolated mold strains on the Sabouraud dextrose slope were subcultured on the Sabouraud dextrose plates using a three-point inoculation technique and incubated at 25°C for 5 days, then, identified macroscopically [28].

### Quantifying AFB_1_ using liquid chromatography-tandem MS

#### Preparation of cheese samples

Ten grams of cheese were mixed with 60 mL acetonitrile and 50 mL hexane. The mixture was homogenized for 5 min using an Ultra Turrax homogenizer (Sigma-Aldrich, Germany then centrifuged at 4000 rpm for 10 min). The sample was filtered, and the final extracts were dried using nitrogen current. The residue was dissolved in 0.2 mL methanol and filtered into an autosampler vial using a 0.2 mm syringe filter (Pall Gelman Sciences, Ann Arbor, MI, USA) [29].

#### HPLC-MS MS procedure

The mass spectrometric analysis was conducted using the Alliance 2690 Separations Module, (Waters Alliance, USA) with 10 mL of the sample injected into the C18 column (3.5 mm, 2.1 x 100 mm), and a guard column of the same phase. The extracts were distilled at a rate of 0.2 mL/min. The initial conditions were water-acetonitrile (75:25), for 16 min, followed by water-acetonitrile (10:90) for 24 min. The column was pre-conditioned with 25% acetonitrile. The HPLC system was linked to a MicroMass Quattro Micro triple-quadrupole mass spectrometer (Micromass Ltd., Manchester, UK) with a positive-mode electrospray ionization probe. The compounds were identified and quantified using the multiple reaction monitoring mode [15].

### Statistical analysis

The data were analyzed using Statistical Package for the Social Sciences (SPSS) V.17 (SPSS, Inc., Chicago, IL, USA) and reported as a percentage, minimum, maximum, and mean±standard error of the mean. The calibration curve and trend line equation were created using Excel. Data obtained from AFM_1_ estimation using ELISA and HPLC were compared using t-test (t) in SPSS to see a statistically significant difference between the two methods’ mean results. If p-value <0.05, the mean values of the two methods were supposed to be significantly different.

## Results

**Figure-1:**
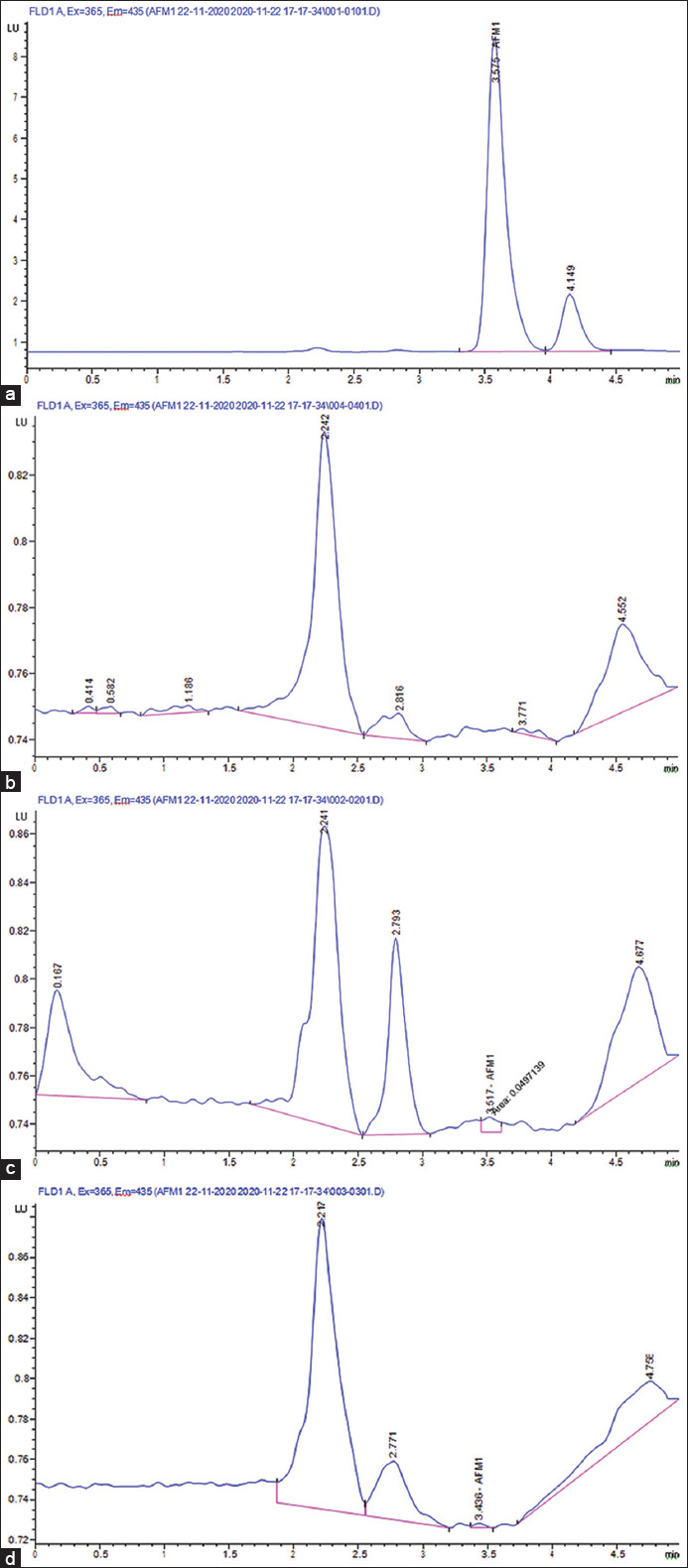
Aflatoxin M1 (AFM_1_) concentrations in the examined samples using high-performance liquid chromatography. (a) AFM_1_ standard (50 ppt). (b) Blank sample. (c) Contaminated sample (26 ppt) and (d) contaminated sample (21 ppt).

Table-1 and Figure-1 show the analyzed results of 115 processed cheese, Ras cheese, and raw milk samples for AFM_1_ contamination. Using the ELISA method; AFM_1_ was found in 82.86% of the tested processed cheese samples with the mean value of 10.77±1.39 ng/kg, whereas all Ras cheese and raw milk samples contaminated with AFM_1_ had a mean value of 51.05±6.19 and 40.27±3.996 ng/kg, respectively. When nearly half of the positive ELISA samples (18 samples from each product) were estimated by HPLC for comparing the obtained mean results using the two methods, consequently, evaluating the sensitivity of the ELISA method as a screening test; 83.33, 100, and 100% of the examined processed cheese, Ras cheese, and raw milk samples was confirmed for contamination with AFM_1_ with mean values of 16.92±2.90, 49.58±7.54, and 49.50±7.23, respectively. The results showed no significant difference between the mean AFM_1_ values estimated using the two methods (t=1.954, p=0.056).

**Table-1:** Statistical analytical results of aflatoxin M_1_ concentration (ng/kg) in the examined samples using ELISA and HPLC methods.

Examined samples		Processed cheese		Ras cheese		Raw milk
ELISA (n=35)	Positive samples no. (%)	29.0 (82.86%)		35.0 (100%)		35.0 (100%)
			Core	Crust	Mix	
	Min.	<5.0	57.46	59.72	<5.00	5.36
	Max.	27.75	100.04	108.14	108.14	103.02
	Mean±SEM	10.77±1.39	75.10±8.43	86.97±8.39	51.05±6.19	40.27±3.996
HPLC method (n=18)	Positive samples no. (%)	15.0 (83.33%)		18.0 (100%)		18.0 (100%)
	Min.	2.0		10.0		10.0
	Max.	30.0		106.0		110.0
	Mean±SEM	16.92±2.90		49.58±7.54		49.50±7.23

*n=Number of examined samples of each food category; processed cheese, Ras cheese, and raw milk. SEM=Standard error of the mean, ELISA=Enzyme-linked immunosorbent assay, HPLC=High-performance liquid chromatography

**Figure-2:**
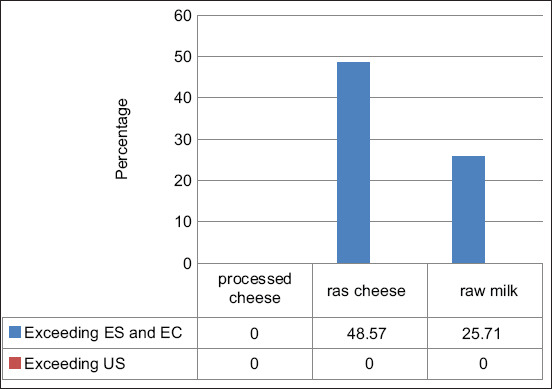
Compatibility of the examined samples with different regulation standards regarding their aflatoxin M_1_ (AFM_1_) content. ES: The Egyptian standards (ES, 7136/2010) [18]. EC: European Commission regulation no. 165/2010 [17]. They indicated that milk and dairy products should not contain AFM_1_ more than 50 ppt. US: United States regulation which established a maximum limit of 500 ppt for milk and milk products [19].

All examined samples were agreed with the prescribed limit of US regulations for AFM_1_. None of the examined processed cheese samples exceeded the recommended safety limits of the EC and the Egyptian regulation for AFM_1_. In contrast, 48.57% and 25.71% of Ras cheese and raw milk samples, respectively, were unacceptable (Figure-2). Our study showed that mold was found in 48.57, 45.71, and 65.71% samples with mean counts of 3.79±3.29, 4.39±4.34, and 4.84±4.29 log CFU/g or mL in the examined processed cheese, Ras cheese, and raw milk samples, respectively (Table-2). 51.43% and 54.49% of the examined processed and Ras cheese samples respectively, were compatible with the Egyptian standards regarding their mold count as illustrated in Table-3.

**Table-2:** Statistical analytical results of mold count (log CFU/g or mL) of the examined samples (n=35).

Examined samples	Positive samples		Min.	Max.	Mean±SEM

	No.	%			
Processed cheese	17	48.57	1	4.4	3.79±3.29
Ras cheese	16	45.71	1.04	5.54	4.39±4.34
Raw milk	23	65.71	1	5.61	4.84±4.29

CFU=Colony-forming unit, SEM=Standard error of the mean

**Table-3:** Compatibility of the examined samples with the Egyptian standards regarding their mold count.

Examined samples	Egyptian standards	Critical limit CFU/g	Compatible samples	

			No.	%
Processed cheese	ES: 999-2/2005 [64]	Nil	18.0	51.43
Ras cheese	ES: 1007-5/2005 [65]	Not>10	19.0	54.49

**Figure-3:**
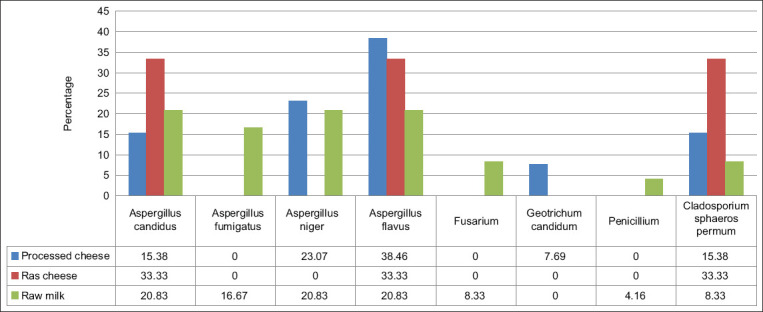
Frequency of occurrence of the isolated mold strains in the examined samples.

**Figure-4:**
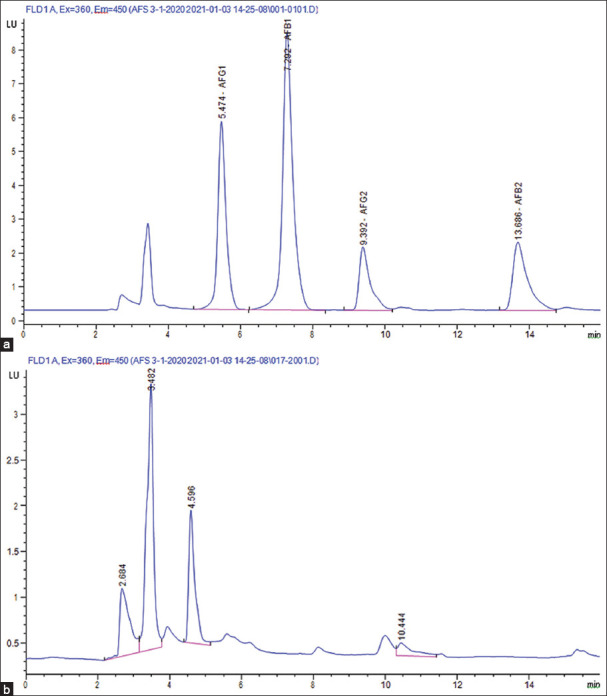
Aflatoxin B_1_ (AFB_1_) results in the examined samples using high-performance liquid chromatography. (a) AFB_1_ standard (5000 ppt). (b) Sample free from AFB_1_.

Figure-3 depicts different mold species isolated from cheese and milk samples. All mold contaminated samples were examined for the presence of AFB_1_ and the toxin was absent (Figure-4). Results of AFM_1_ and AFB_1_ verified that there is no direct relationship between the presence of AFM_1_ and AFB_1_ in milk and milk products.

## Discussion

### Prevalence of AFM_1_ in the examined samples

Mold contamination was considered a quality issue rather than a threat to food safety, later some species ability to produce toxigenic mycotoxins made it a threat to public health, as they can cause human food poisoning outbreaks in addition to their carcinogenic effect. Consequently, the level of mold contamination, identification of the predominant mold species, and the level of mycotoxins are essential for determining the quality and safety of milk and dairy products [30-32].

Some mold spores produce AFB_1_, which directly reaches humans through contaminated food or as a metabolic residue in food of animal origin. The liver metabolizes AFB_1_ into AFM_1_ and is secreted in the milk. AFM_1_ is stable in raw milk and processed milk products, making milk and dairy products the primary vehicle for introducing AFM_1_ into the human diet. AFM_1_ is hepatotoxic, mutagenic, and carcinogenic; its carcinogenicity is nearly 2-10% greater than AFB_1._ This made the IARC to transfer AFM_1_ from Group 2B (possible) human carcinogen to Group 1 (proven) carcinogen [33].

The mean AFM_1_ content in processed cheese was similar to that obtained by Ahmed *et al*. [22] and Tahoun *et al*. [34] who detected AFM_1_ with a mean value of 24.53±3.91 ng/kg, while higher values were reported by Amer and Ibrahim [35] and El-Kest *et al*. [36].

Findings related to Ras cheese were in agreement with Amer and Ibrahim [35] and Aiad and Abo Ei-Makarem [11] who found that the mean AFM_1_ value in the examined Ras cheese was 56.048±6.29 ng/kg; while higher than Hosny *et al*. [37], Ahmed *et al*. [22], and Younis *et al*. [38] who reported that the examined samples of Ras cheese were contaminated with AFM_1_ with a mean value of 15 ng/kg. The results were lower than those cited by Nassib *et al*. [39] and Abdel All *et al*. [40].

The mean values of AFM_1_ in the core and crust samples of Ras cheese were 75.10±8.43 and 86.97±8.39, respectively, with no statistically significant difference. These results were parallel to those recorded by Bahout and El-Shawaf [41] who examined 50 Ras cheese and found that the mean AFM_1_ in cheese surface (at depth <5 mm) (6660 ng/kg) was nearly similar to the cheese interior samples (at depth >5 mm) (6540 ng/kg).

However, similar findings of AFM_1_ in raw milk samples were reported by. Yilmaz and Altinci [25], Tahoun *et al*. [34] and Younis *et al*. [38]. In comparison, lower detectable levels of AFM_1_ were recorded by Lee *et al*. [42] and Elzupir and Elhussein [43]. A higher incidence of AFM_1_ was recorded by Kirino *et al*. [44], Nadira *et al*. [45], and Kagera *et al*. [46].

Due to the hazardous nature of AFM_1_ along with its extreme thermal resistance, most countries established legal regulations for AFM_1_ in raw milk and dairy products with an admissible limit, which varies from 50 ng/kg recommended by EC regulation and the Egyptian standard [17,18] to 500 ng/kg established by the Codex Alimentarius Commission and National Agency for Food and Drug Administration. Our study showed that all positive processed cheese, Ras cheese, and raw milk examined samples agreed with the mentioned limit of the US regulations. None of the processed cheese examined samples exceeded the prescribed safety limits of the EC and the Egyptian regulation. In contrast, 48.57% and 25.71% of Ras cheese and raw milk examined samples, respectively, were unacceptable.

The acceptability of this toxin in milk and dairy products was studied worldwide, from the results that exceeded the limit set up by many countries of 500 ng/kg [40,43]. Alternatively, other studies exceeded the limits imposed by the EC [20,46,47].

The contamination level of milk and milk products with AFM_1_ varies widely according to dairy feed quality, environmental factors, variation in the original milk contamination, cheese production technologies, type of cheese, extraction, and analytical methods, including regulatory limits for AFM_1_ in animal feeds, milk, and dairy products [11,48]. In addition, when cheeses were compared to the milk from which they were made, soft cheeses had a 3-fold greater AFM_1_ concentration while hard cheeses had a 5-fold greater concentration due to the preferred affinity of AFM_1_ for casein fraction [49].

The most commonly used analytical methods for the quantification of AFM_1_ in milk include TLC, HPLC with a fluorescence detector (HPLC-FL), and the ELISA [50]. Notwithstanding its extensive and time-consuming sample preparation that necessitates the use of numerous chemical solvents, HPLC-FL is currently the most accurate method [51-53]; however, ELISA gives quick and sensitive results, cost-effective, and requires small sample volumes and fewer preparation procedures. Therefore, ELISA can be a reliable alternative to HPLC-FL and a preferred method at the routine level and in research studies [53]. HPLC-FL as a reference method is used for confirming the obtained ELISA results. Especially due to cross-reaction interferences, particularly at concentrations <50 ng/L, the ELISA method may not be completely reliable as it is resulting in false-positive or false-negative results [53,54].

There is statistically no significant difference between the mean AFM_1_ values estimated using ELISA and HPLC methods when nearly half of the positive ELISA samples of processed cheese, Ras cheese, and raw milk were reexamined by HPLC; hence, the mean AFM_1_ was 36.81±4.25 and 37.98±4.31**-**ng/kg by ELISA and HPLC, respectively (p>0.05). These results are comparable to those reported by Mwanza *et al*. [50] and Maggira *et al*. [53]. The obtained results confirm the high degree of ELISA sensitivity and accuracy.

The examined samples revealed contamination with a carcinogen, which remains relatively stable after pasteurization, storage, and preparation of dairy products and poses a serious threat to children and the elderly who consume it, thus, emphasizing the importance of lowering AFM_1_ levels in milk to the absolute minimum. Therefore, continuous monitoring surveys should be considered in this regard, including the feedstuff ration being kept away from fungal contamination and checked regularly to be free from AFB_1_ contamination. Further studies and applica­tion of new or modern technologies for AFM_1_ detoxi­fication is necessary [34,55].

### Total mold count in the tested samples

Contamination of mold in some of the examined samples could be attributed to the unhygienic milking procedures and equipment used for milking, inadequate refrigeration during storage and distribution, inadequate sanitation during manufacturing and ripening, warm weather, and poor personal hygiene, moreover, air and sackcloth packaging of Ras cheese are considered major sources of fungal contamination. The presence of mold in processed cheese indicates post-processing contamination or the survival of mold spores [30,56,57].

The results of processed cheese and Ras cheese samples were similar to those reported by Mohamed *et al*. [57]. Higher results were obtained by Abdel-Salam and Soliman [58] who found that the mean mold counts of processed cheese and Ras cheese were 5.83±5.80 and 5.56±5.40 log CFU/g, respectively, and Mohamed *et al*. [31] who examined Ras cheese and found that the mean mold count was 4.85 log CFU/g. While lower results were obtained by Hameed [59] who examined processed cheese and found that the mean mold count was 1.23±0.4 log CFU/g.

Concerning raw milk samples, mold count agreed with that reported by Gurmessa [60] and Amentie *et al*. [61], while the mold count was lower than that reported by El-Diasty and El-Kaseh [62] and higher than Talukder *et al*. [63].

On matching the aforementioned results with the Egyptian specification, it was clear that 48.57% and 45.71% of the examined processed and Ras cheese did not match with the Egyptian standards [64,65] for mold count, respectively. These high counts may result in severe economic losses due to the associated visible signs of spoilage as discoloration and off-flavor, with the possibility of mycotoxin production [30,56].

The isolated mold stains in the study were in agreement with those obtained by other researchers; Elbagory *et al*. [66] and Seddek *et al*. [67] who showed that *Aspergillus* was the most predominant isolated mold from Ras cheese and represented by *Aspergillus* *flavus*, *Aspergillus* *niger*, *Aspergillus ustus*, and *Aspergillus* *fumigatus*. Another study conducted by Mohamed *et al*. [57] who showed that the most prevalent mold isolates from processed and Ras cheeses were Penicillium followed by *Aspergillus*.

These findings highlight the importance of employing more stringent sanitary practices to reduce the risks associated with the fungal contamination of milk and milk products, thereby improving the quality as well as the safety of these products; regulatory intervention, including microbiological standards, enhanced sanitation, and food safety programs, should be developed. Biopreservation and novel packaging are also needed to reduce the incidence of mold spoilage in dairy products [68,69].

### Incidence of AFB_1_ in the contaminated samples with mold

The presence of AFB_1_ in milk and milk products may result from ingestion of feedstuffs containing AFB_1_ that the cow liver has not wholly metabolized to AFM_1_, therefore, AFB_1_ is found in milk, as well as the contamination of cheese with mold spores that produce AFB_1_ during processing and storage due to the lack or inadequate hygienic measures applied [70].

The absence of AFB_1_ in all examined samples was in agreement with the Egyptian regulations, which stated that AFB_1_ should be absent in milk and dairy products [18] and the Directive 2003/100/EC of the European Commission establishing a maximum AFB_1_ content of 5000 ng/kg in milk and cheese [71]. Results of AFM_1_ and AFB_1_ verified that there is no straight relationship between AFM_1_ and AFB_1_ present in milk and milk products.

Similar results were also reported by Montagna *et al*. [72] and Embaby *et al*. [73] who stated that all examined buffalo milk cheese samples were consistently negative for AFB_1_. In contrast, positive results were recorded by Abou Ayana *et al*. [74] and Mao *et al*. [75].

## Conclusion

The detection of AFs in food remains an essential subject in a food safety investigation. The current study revealed that processed cheese, Ras cheese, and raw milk samples were contaminated with AFM_1_. In addition, there was statistically no significant difference between AFM_1_ levels in the core and crust parts of the tested Ras cheese samples (p>0.05). AFM_1_ levels in processed cheese did not exceed the maximum limits set by the Egyptian standards, while 48.57% and 25.71% of Ras cheese and raw milk samples, respectively, were above the imposed limit. The examined samples were contaminated with toxigenic mold strains. However, they did not show AFB_1_ contamination., AFB_1_ results verified no straight relationship between AFM_1_ and AFB_1_ presence in milk and dairy products. The results of the comparative evaluation of ELISA and HPLC methods demonstrated a satisfactory correlation between both methods with no significant difference (p>0.05). These results recommend that the rapid ELISA method can be used for routine analysis, while HPLC is still the gold standard for confirmation. Overall, AFM_1_ prevalence is considered a significant risk to human health; as a result, all milk products must be kept within the allowed limit. Moreover, integrated surveillance programs should be implemented to continuously monitor AFM_1_ levels in milk and dairy products. A novel method should be conducted and applied to ensure the safety of milk and milk products for human consumption by avoiding or reducing the presence of these toxic contaminants.

## Authors’ Contributions

RME: Conceptualization, methodology, and drafted the manuscript. RSH: Conceptualization, revised the manuscript, and visualization. NIMK: Visualization, methodology, and supervision**.** KMF: Investigation, methodology, visualization, and revised the manuscript. LIA: Conceptualization, methodology, original draft preparation, investigation, and revised the manuscript. All authors read and approved the final manuscript.
